# A Preliminary In Vitro Study on the Efficacy of High-Power Photodynamic Therapy (HLLT): Comparison between Pulsed Diode Lasers and Superpulsed Diode Lasers and Impact of Hydrogen Peroxide with Controlled Stabilization

**DOI:** 10.1155/2016/1386158

**Published:** 2016-08-18

**Authors:** Gianluigi Caccianiga, Marco Baldoni, Carlo Angelo Ghisalberti, Alessio Paiusco

**Affiliations:** ^1^School of Medicine and Surgery, University of Milano-Bicocca, Milan, Italy; ^2^Faculty of Medical Sciences, LUDES HEI Foundation, Malta; ^3^Department of Biomedical Sciences for Health, University of Milan, Milan, Italy

## Abstract

*Aim*. In periodontology lasers have been suggested for the photodynamic therapy (PDT): such therapy can be defined as the inactivation of cells, microorganisms, or molecules induced by light and not by heat. The aim of this study was to evaluate results of PDT using a 980 nm diode laser (Wiser Doctor Smile, Lambda SPA, Italy) combined with hydrogen peroxide, comparing a pulsed diode laser (LI) activity to a high-frequency superpulsed diode laser (LII).* Materials and Methods*. Primary fibroblasts and keratinocytes cell lines, isolated from human dermis, were irradiated every 48 h for 10 days using LI and LII combined with SiOxyL^+^ *™* Solution (hydrogen peroxide (HP) stabilized with a glycerol phosphate complex). Two days after the last irradiation, the treated cultures were analyzed by flow cytofluorometry (FACS) and western blotting to quantify keratin 5 and keratin 8 with monoclonal antibodies reactive to cytokeratin 5 and cytokeratin 8. Antimicrobial activity was also evaluated.* Results*. Both experimental models show the superiority of LII against LI. In parallel, stabilized HP provided better results in the regeneration test in respect to common HP, while the biocidal activity remains comparable.* Conclusion*. The use of high-frequency lasers combined with stabilized hydrogen peroxide can provide optimal results for a substantial decrease of bacterial count combined with a maximal biostimulation induction of soft tissues and osteogenesis.

## 1. Introduction

Laser versatility in dentistry, alternatively to or combined with scalpels, rotary instruments, and other surgical protocols, ensures less painful and invasive treatments, being also more precise and efficient and showing a high hemostatic control.

The benefit of this approach has been underlined for more than a decade [1]. Combined with traditional instruments, lasers can be used in all dentistry areas: oral surgery, implantology, periodontology, conservative dentistry, dental aesthetics, and endodontics, provided proper integrations of the application protocols are foreseen [2].

In periodontology lasers have been suggested for the photodynamic therapy (PDT): such therapy can be defined as the inactivation of cells, microorganisms, or molecules induced by light and not by heat.

PDT requires a light source (laser), a photosensitizer (a substance containing oxygen), and oxygenated tissues. Oxygen in fact is the crucial molecule for performing PDT. “Photodynamic” implies the application of luminous photonic dynamics on biological molecules [2–5].

The mechanism of action of PDT foresees the interaction of light with the dye the target tissues have been imbibed with. The dyed molecules adapt to the bacterial membrane of microorganisms [6, 7]. The laser light activates the dye molecule or photosensitizer, while the resulting reaction with oxygen releases triplet oxygen with 2 unpaired, parallel-spin electrons [8, 9]. Given the coupling of 2 unpaired, opposite-spin electrons, the interaction between triplet oxygen and laser energy results in the formation of singlet oxygen, which determines the oxidation of the lipid membrane of bacteria and their cell death [2, 10–13].

To date only lasers with high penetration depth (600 to 1100 nm) have been taken into consideration for PDT, since they are scarcely absorbed by water and hydroxyapatite and in particular diode lasers. Thanks to such low absorption level, wavelengths comprised within this range can penetrate in tissues up to 2 cm. This can be especially suitable for the treatment of pathologies characterized by high bacterial dissemination, like periodontal diseases, whereas mechanical treatment protocols can only act on the directly treated surfaces, such as the hard tissues of the tooth (cement and dentine) and the hard and soft tissues of the periodontium comprised within the treatment site. The possibility of a deeper penetration could be useful to eradicate those bacteria that are involved in the pathology but that are not necessarily contiguous with the sick tooth.

Under normal setting conditions, diode lasers with power beyond 2 Watts (HLLT: High Level Laser Therapy) show a high thermal effect [14]; that is the reason why research has basically tested low-power diode lasers (LLLT: Low Level Laser Therapy) with energy pulses comprised within milliseconds (pulsed lasers) or continuously emitted energy pulses that cannot produce a significant temperature rise (above 45 centigrade degrees) and that are managed together with dyed photosensitizers, with typical absorption ranges in long wavelength bands.

However, it has been noticed that classic PDT is only partially effective in diseases showing deep bacterial infiltration [15]. This can be ascribed to the scarce peak power applied, below 2 Watts, as well as to the scarce penetration capacity of the laser light in tissues imbibed with photosensitizer, with a biocidal effect that can only be limited to the external and/or superficial areas in nonsurgical or open surgeries, for example, in the surgical treatment of peri-implantitis.

Although it does not show significant advantages in respect to surgery, classic PDT with pulsed or continuous LLLT and blue photoactivators seems to have a positive effect on inflammatory indexes [16].

The various photosensitizing chromophoric agents have been compared on* S. mutans* strains as an oral biofilm model. Toluidine blue ortho (TBO) was the only one able to substantially reduce a bacterial load of 3 Log, while others, such as methylene blue (MB), malachite green (MG), eosin (EOS), erythrosine (ERI), and rose Bengal (RB), proved to be less efficient [17].

The nonsurgical periodontal therapy combining a 980 nm laser with hydrogen peroxide is gaining more and more consensus in clinical dental practice as shown in Rey protocol [18, 19].

**Figure 1:**
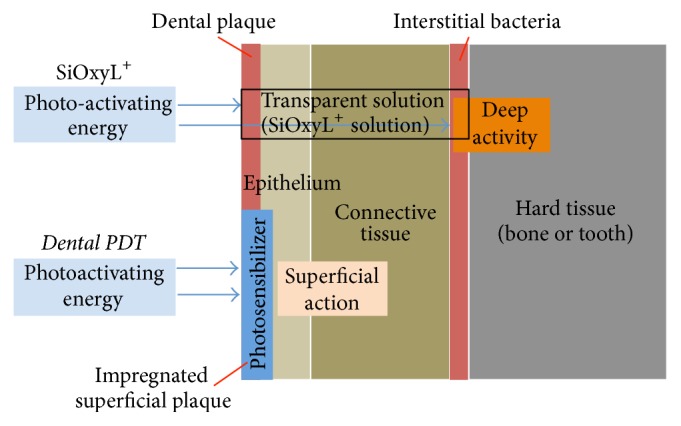
Benefits of the transparent photosensitizer.

The benefits of hydrogen peroxide as opposed to classic photodynamic therapy (PDT) performed with photoactivating agents with absorption within the visible band consist in a higher bioavailability and deeper penetration in the biofilms as well as in the scarce interference in respect to the irradiation performed (Figure 1) [18].

The aim of this study was to evaluate the efficacy of low-frequency diode lasers (LI) compared to high-frequency lasers (LII), related also to effects of stabilizers contribution on hydrogen peroxide properties. The advantage to use LII could be to have more efficacy than LI in order to deliver singlet oxygen when laser meets hydrogen peroxide (more than 7000 times per second compared to 50/500 impacts). High frequency could improve the activity of the impacts inside the soft tissues and the efficacy of decontaminating effects of HLLT.

## 2. Materials and Methods

All tests were made by the same investigator.

### 2.1. Reagents

The 3% hydrogen peroxide stabilized with 200 ppm acetanilide (catalogue number 323381, HP-C), the 30% nonstabilized hydrogen peroxide, the sodium phosphate monobasic hexahydrate, and the glycerol phosphate disodium salt hydrated were purchased from Sigma-Aldrich (Milan, Italy).

The hydrogen peroxide solution with physiologic stabilization (HP-GC) is prepared by diluting with bidistilled water the nonstabilized H_2_O_2_ solution in 1 : 10 v/v and by dissolving the triad glycerin/monosodium phosphate/glycerol phosphate disodium with a 50/7/1 molar ratio in a quantity equivalent to 3.7% p/p of the solution.

### 2.2. Irradiation Sources

The irradiation sources were as follows:

(i) (LI) 980 nm diode laser (Wiser Doctor Smile, Lambda SpA, Italy) with 400 micron fiber, set to 2.5 Watts, (mean energy 0.625 W), and *T*_ON_ 5 milliseconds and *T*_OFF_ 15 milliseconds, with 50 Hertz frequency and application time 50 seconds. Operator used the same way to irradiate all samples.
(ii) (LII) High-frequency laser (Wiser Doctor Smile, Lambda SpA, Italy), set to “decontamination,” with 400-micron fiber, with characteristics as shown under Table 1 and application time 50 seconds (mean energy 0.5 W, frequency > 7000 Hz).

### 2.3. Evaluation of the Biostimulating Effect

Primary fibroblasts and keratinocytes cell lines (Matched Set-Cryopreserved Dermal Fibroblasts and Keratinocytes, Tebu-Bio*™*) isolated from human dermis are placed, respectively, in the culture media Euroclone*™* and Tebu-bio (Human Adult Keratinocyte Growth Medium KM-2).

The culture of the lines is confluent-type (70–80% min) in a 1 : 1 mix of the two culture media (final FBS + 5%, named “A”). In racks equipped with 12 1 cm wells, single-layer fibroblasts (0.5 × 105 cells/well) and keratinocytes (1 × 10^5^ cells/well) are seeded or grown in an “organotypic” coculture.

0.3 mL of HP-GP solution, HP-C solution, or distilled water is added, respectively, to the cultures (control). Culture media are changed every 48 hours matching the irradiation treatment that is performed with LI and LII at 48 h intervals for 10 days.

The interval of 48 h in vitro is the minimum but also sufficient to allow the fibroblasts and keratinocytes to double their population. In fact, the average time of cell-doubling for keratinocytes is 40.5 h, something more for fibroblasts. In this way, the new generation of the cells has the time to express and produce keratin 5 and keratin 8, proteins of reinforcement of the junctional epithelium, stimulated by moderate stress factors.

According to common protocols in an vitro study, two days after the last irradiation, the treated cultures are analyzed by flow cytofluorometry (FACS) and western blotting to quantify keratin 5 and keratin 8 with monoclonal antibodies reactive to (cyto)keratin 5 and (cyto)keratin 8 (KRT 5/8, Antibodies-Online*™*).

### 2.4. Verification of the Biocidal Activity

The test is performed with current methods [18] on cultures of typical strains causing infections in the oral cavity.

Selection of the pathogenic strains is as follows:

- *Haemophilus actinomycetemcomitans* CIP 52103T (“HA”).
- *Bacteroides forsythus* CIP 105219T (“BF”).
- *Porphyromonas gingivalis* CIP 103683T (“PG”).
- *Micromonas micros* CIP 105294T (“MM”).
- *Fusobacterium nucleatum* CIP 101130T (“FN”).

A 30 *μ*L suspension for each strain is placed in 1.5 mL Eppendorf tubes with 5% of culture medium and is treated with LI and LII with 10 s irradiations along the test tube, specifically 5 s of vertical motion and 5 s of rotary motion. Washing is performed with 1 part of hydrogen peroxide solution in 2 parts of culture solution with a 3 min contact time, checking for any temperature increase. At the end the population density is measured in CFUs (colony-forming units).

### 2.5. Statistical Analysis

All experiments were performed 3 times. Differences between groups were determined by ANOVA.* p* values of less than 0.05^*∗*^ are considered significant. Data are given as mean ± standard deviation (s.d.). All statistical analyses were performed employing the statistical algorithms in Microsoft*™* Excel*™* per Mac, release 14.6.5.

## 3. Results

**Figure 2:**
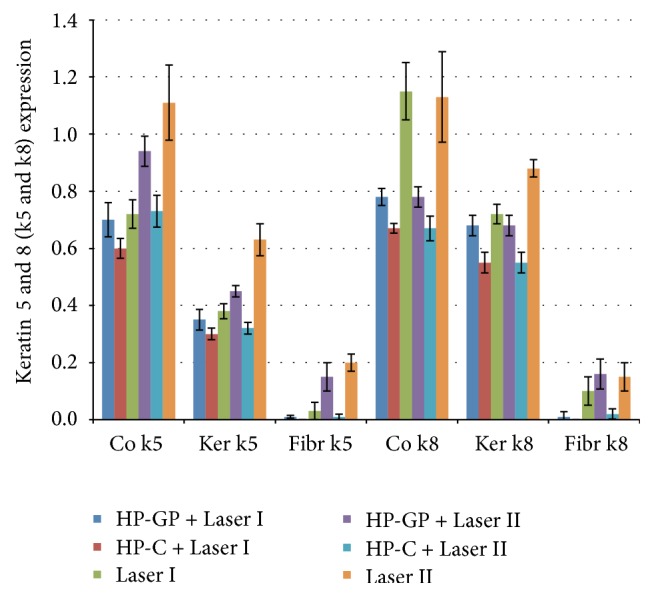
Cytostimulating activity. Western blot expression of keratin 5 (k5) and keratin 8 (k8) from organotypic coculture (Co), keratinocytes (Ker), and fibroblasts (Fibr) after 6x irradiation with (blue bars) Laser I alone; (red bar) Laser I with hydrogen peroxide-glycerol phosphate complex (HP-GP); (green bar) Laser I with common hydrogen peroxide (HP-C); (violet bar) Laser II alone; (light blue bar) Laser II with hydrogen peroxide-glycerol phosphate complex (HP-GP); and (orange bar) Laser I with common hydrogen peroxide (HP-C) Laser I. Experiments were performed 3 times. Data are given as mean ± standard deviation (s.d.).

**Figure 3:**
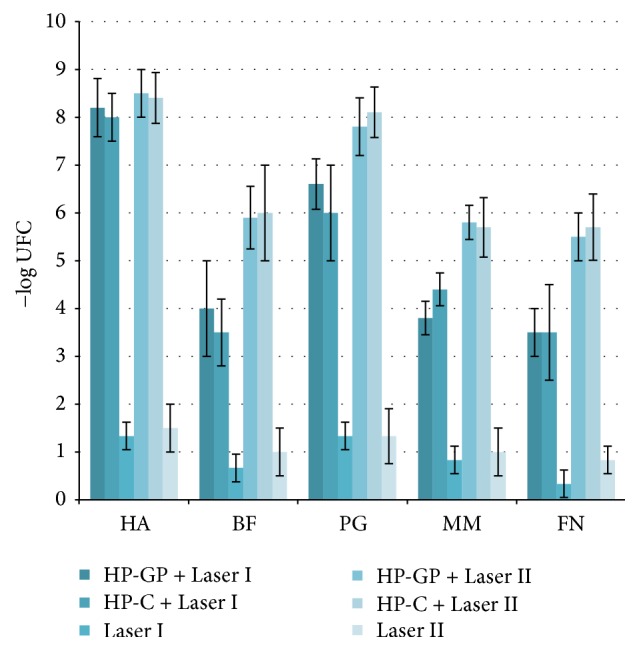
Sanitizing activity on the main bacteria involved in periodontal diseases. Decrease of pathogenic bacteria expressed as Unit Forming Colonies (UFC) of* Actinobacillus actinomycetemcomitans* (HA),* Bacteroides forsythus* (BF) or* Tannerella forsythensis*,* Porphyromonas gingivalis* (PG),* Micromonas micros* or* Peptostreptococcus micros* (MM), and* Fusobacterium nucleatum* (FN) upon treatment with Laser I + HP-GP; Laser I + HP-C; Laser I; Laser II + HP-GP; Laser II + HP-C; and Laser II. Experiments were performed 3 times. Data are given as mean ± standard deviation (s.d.).

**Figure 4:**
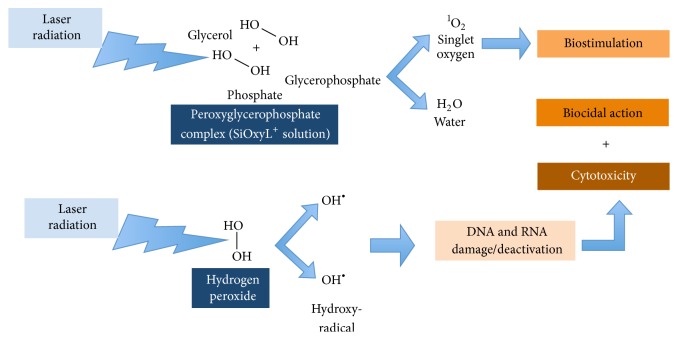
Mechanism of action of SiOxyL^+^ HLL Technology (High-Frequency Diode Laser Wiser and SiOxyL^+^ solution).

The comparative evaluation of biostimulation data (Figure 2) and biocidal efficiency data (Figure 3) indicates a higher efficiency of high-frequency lasers (LII) in respect to diode lasers (LI). The same experimental kit shows the effects of the stabilizers contribution on hydrogen peroxide properties. The use of hydrogen peroxide in a glycerol phosphate complex (HP-GP) provides a substantial decrease of the bacterial load that can be compared to that of the common hydrogen peroxide (HP-C) and to the minimization of the cytotoxic impact thanks to the particular physiologic-like composition (Figure 4).

In more detail, the expression of keratin 5–8 upon repeated LI exposure was slightly higher in the presence of hydrogen peroxide-glycerol phosphate complex (HP-GP) compared to common hydrogen peroxide (HP-C) in keratinocytes and fibroblast cultures and cocultures. The absence of HP as in control group (Laser I) slightly improved biostimulation, although the differences were not statistically significant (*p* = 0.3646). Overall higher biostimulation was attained from the data with LII, yet the same pattern is observed. HP-GP performed better than HP-C, while Laser II produces a higher expression of keratin 5 to keratin 8. Again, the difference between tests and control group was not statistically significant (*p* = 0.0415). Conversely, high sanitizing efficiency in the in vitro model was attained by either glycerol phosphate-stabilized hydrogen peroxide or common hydrogen peroxide in conjunction with Laser I/II, which afforded almost negligible decrease of pathogenic contamination (Figure 3).

## 4. Discussion

PDT performed with pulsed or continuous LLLT seems to show clear efficacy limits due to the following reasons:

- The very low power (below 1 Watt) cannot ensure a proper bactericidal efficacy on microorganisms that are responsible for periodontal diseases.
- The laser penetration capacity is limited, due to the energy absorbed in tissues imbibed with dyed photosensitizer [18, 20, 21].

Nonetheless, LLLT shows a good biostimulation effect: the purpose of laser-assisted biostimulation is to stimulate the activity of the cells designated to the regeneration of tissues [18, 20, 21] lost because of the aggression of oral pathogens. Moreover, laser biostimulation significantly activates the proliferation and differentiation of adult mesenchymal stem cells in the line required in the defect area caused by the periodontal disease [17, 22–24].

With HLLT pulsed (LI) the very long pulse time (within the milliseconds range) can emit frequencies that do not exceed 7000 Hz; this reduces the activation capacity of the hydrogen peroxide's derivate (SiOxyL^+^ solution) and the correspondent release of singlet oxygen, which is crucial to ensure a decontaminating effect on microorganisms.

The use of HLLT with the “SiOxyL^+^ HLL Technology,” a superpulsed laser, goes beyond the limits of conventional PDT, since it allows combining the high peak power required to eliminate pathogens in the oral cavity (higher than 2 W) with a low mean power (below 0.8 W) that is suitable to promote laser-assisted biostimulation, whereas temperature does not exceed 45°C and remains inside the range of tissue vasodilation.

Moreover, a frequency higher than 7 KHz as determined by the pulse length in microseconds (superpulsed laser) triggers thousands of activation events per second of the SiOxyL^+^ solution, resulting in continuous production of singlet oxygen that causes the cell death of the pathogenic bacteria that are responsible for infection diseases in the mouth [18, 20–22].

The use of diluted solutions of hydrogen peroxide combined with a 980 nm laser seems able to provide for a deep sanitization [18, 20]. Hydrogen peroxide is characterized by a moderate antibacterial capacity, and the laser increases its efficiency thanks to the photodynamic action due to the activation of peroxide. In fact, the transfer of energy from the laser to the H_2_O_2_ molecule results in its homolytic scission to OH^−^ (hydroxyl-radical) or its decomposition to H_2_O and ^1^O_2_ (singlet oxygen).

The limits of this method, if any, are to be ascribed to the hydrogen peroxide quality, specifically to the type of stabilizers that are required to avoid the decomposition of the aqueous solution of H_2_O_2_. When irradiated, stabilizers such as colloidal tin, silver nitrate, organophosphates, nitrates, and acetonitrile may generate free radicals and have therefore irritating effects.

It seemed appropriate to further increase the balance between antiseptic and regenerating properties. Laboratory methods were employed in order to evaluate a hydrogen peroxide composition with the best ratio between stability, antibacterial action, and low impact (nonnegative contribution) to laser biostimulation.

Hydrogen peroxide at 10 volumes 3% has no cytotoxic effect on human cells, as can instead occur with peroxide at 20 volumes; however, biostimulation implementation can be an important aim in therapies using HLLT Technology.

The addition of a complex containing peroxide-glycerol phosphate is based on the fact that this component promotes fibroblasts cellular vitality. So composition of common hydrogen peroxide was modified evaluating the adjunctive benefits of this complex, creating SiOxyL^+^ solution.

Tests of cell viability, made on fibroblasts and keratinocytes, effectively showed an activity implementation of these cells compared to the use of common hydrogen peroxide at 10 volumes 3%. Nowadays there are no similar studies published in literature, but some in vivo studies performed on periodontal disease and bone regeneration showed the excellent tissue response to HLLT performed with SiOxyL^+^ solution.

## 5. Conclusions

A solution to optimize such therapies seems to be the use of high-frequency lasers (LII) combined with hydrogen peroxide stabilized with glycerol phosphate complex (HP-GP) that provides optimal results for a substantial decrease of the bacterial load combined with a maximal biostimulation induction of soft tissues and osteogenesis.

## Figures and Tables

**Table 1:** High-frequency laser technical specifications.

Laser source	Semiconductor
Wavelength	980 nm
Max power	7 W
Power resolution	Digital 0.1 W to 7.0 W, resolution 0.1 W

Available pulses	Peak power > 2 W, mean power < 0.8 W, frequency > 8 KHz
Settings allowed	Decontamination, regeneration, peri-implantitis, light biostimulation, medium biostimulation
